# Effect of hormone replacement therapy on amyloid beta (Aβ) plaque density in the rhesus macaque amygdala

**DOI:** 10.3389/fnagi.2023.1326747

**Published:** 2024-01-11

**Authors:** Maria-Luisa Appleman, Jeremy L. Thomas, Alison R. Weiss, Benjamin I. Nilaver, Rita Cervera-Juanes, Steven G. Kohama, Henryk F. Urbanski

**Affiliations:** ^1^Division of Neuroscience, Oregon National Primate Research Center, Beaverton, OR, United States; ^2^Department of Physiology and Pharmacology, Atrium Health Wake Forest Baptist Medical Center, Winston-Salem, NC, United States; ^3^Division of Reproductive & Developmental Sciences, Oregon National Primate Research Center, Beaverton, OR, United States; ^4^Department of Behavioral Neuroscience, Oregon Health & Science University, Portland, OR, United States

**Keywords:** aging, Alzheimer’s disease, amyloid plaques, hormone replacement therapy (HRT), rhesus macaque, menopause

## Abstract

**Background:**

Amyloid beta (Aβ) plaque density was examined in the amygdala of rhesus macaques, to elucidate the influence of age, diet and hormonal environment.

**Methods:**

Luminex technology was used to measure cerebrospinal fluid (CSF) concentrations of Aβ_40_ and Aβ_42_ across three decades, while immunohistochemistry was used to examine Aβ plaque density in the amygdala.

**Results:**

Aβ_40_ was found to be the predominant isoform of Aβ in the CSF, but neither Aβ_40_ or Aβ_42_ concentrations showed an age-related change, and the ratio of Aβ_42_ to Aβ_40_ showed only a marginal increase. Significantly fewer Aβ plaques were detected in the amygdala of old ovariectomized animals if they received estradiol HRT (*p* < 0.001); similar results were obtained regardless of whether they had been maintained on a regular monkey chow for ∼48 months or on a high-fat, high-sugar, Western-style diet for ∼30 months.

**Conclusion:**

The results demonstrate that HRT involving estrogen can reduce Aβ plaque load in a cognitive brain region of aged non-human primates. The results from this translational animal model may therefore have clinical relevance to the treatment of AD in post-menopausal women, whether used alone, or as a supplement to current pharmacological and monoclonal antibody-based interventions.

## Introduction

Amyloid beta protein (Aβ) has long been considered a pathological marker of Alzheimer’s disease (AD) and related dementias, and is generally found in pathological plaque deposits as two major forms, Aβ_40_ and Aβ_42_ (De-Paula et al., 2012; Alzheimer’s Dement, 2023). However, many subjects with extensive Aβ plaques do not show obvious dementia (Chételat et al., 2011, 2012). Consequently, it is unclear if Aβ plaques play a causal role in triggering AD or if they simply reflect a secondary response to damage and/or inflammation resulting from insults, such as bacterial/viral infections and pollutants. Nevertheless, there is much support for an amyloid cascade hypothesis, which postulates that abnormal accumulation of Aβ plaques within the brain initiates a sequence of events that ultimately lead to AD dementia (Karran et al., 2011; Selkoe and Hardy, 2016; Tolar et al., 2020). Based on this hypothesis many pharmaceutical interventions have targeted reduction of Aβ, or facilitation of its clearance, but most have failed in Phase III clinical trials. More encouragingly, antibody-based interventions have efficacy at reducing Aβ accumulation, and have even received fast-track FDA-approval (Sevigny et al., 2016; Tian Hui Kwan et al., 2020; Avgerinos et al., 2021; Decourt et al., 2021; Shi et al., 2022; Mead and Fox, 2023; van Dyck et al., 2023). On the other hand, the long-term safety of these pharmacological and immunological interventions is unclear, nor are individual differences in treatment response. Consequently, there is a need for additional interventions that can help to reduce Aβ load within the brain, especially in those areas involved in cognitive functions such as the prefrontal cortex, hippocampus and amygdala.

One such intervention that has previously been proposed involves estrogen hormone replacement therapy (HRT). This stems from the observation that the risk of developing AD is greater in women than in men (Viña and Lloret, 2010; Laws et al., 2018; Scheyer et al., 2018) and that HRT can improve cognitive function after menopause (Tang et al., 1996; Kawas et al., 1997; Yaffe et al., 1998; Zandi et al., 2002; Yaffe, 2003). On the other hand, some clinical studies have failed to show any beneficial effects of HRT (Seshadri et al., 2001) or have suggested that HRT increases the risk for developing AD, depending on when the HRT is initiated (Shumaker et al., 2003; Shao et al., 2012; Pourhadi et al., 2023). Consequently, the therapeutic potential of supplementary estrogen for AD is unclear as is the contribution of the sex-steroid environment to the development of Aβ plaques. Slow progress in resolving this issue stems partly from a paucity of experimental animal models, other than transgenic rodents, in which to examine environmental factors that contribute to the formation of Aβ plaques. In this regard, the rhesus macaque represents a valuable animal model with more immediate translational potential to the clinic (Messaoudi et al., 2011; Colman, 2018; Stonebarger et al., 2021). Like humans, these long-lived non-human primates (NHPs) show similar brain organization and development. Also, like women, female rhesus macaques show menstrual cycles and eventually undergo menopause (Gilardi et al., 1997; Downs and Urbanski, 2006: Sorwell and Urbanski, 2013; Luna et al., 2020). Furthermore, old rhesus macaques, like elderly humans, progressively express Aβ plaques within the brain (Heilbroner and Kemper, 1990; Martin et al., 1991; Uno, 1993; Sloane et al., 1997; Stonebarger et al., 2020). A major advantage of rhesus macaque studies, however, is that the animals can be maintained under tightly controlled environmental conditions and their brains can be obtained with a zero post-mortem interval.

We previously observed that estradiol hormone replacement therapy (HRT) lowered gene expression of amyloid precursor protein (APP) in the dorsal raphe nucleus of old female rhesus macaques (Bethea et al., 2016) and more recently showed that HRT significantly affects the transcriptome of the amygdala (Cervera-Juanes et al., 2022); specifically, pathway enrichment analysis identified inhibition of neuro-inflammation in the amygdala with HRT, whereas amyloid processing was enriched. This suggests that HRT may exert a protective effect against neuro-inflammation and Aβ deposition within cognitive brain regions such as the amygdala, and could explain why old ovariectomized rhesus macaques show cognitive benefits when subjected to estradiol HRT (Rapp et al., 2003; Tinkler and Voytko, 2005; Lacreuse, 2006; Voytko et al., 2008; Kohama et al., 2016).

Consequently, the aim of the present study was to examine age-related development of Aβ plaques in the rhesus macaque brain; specifically, in the amygdala, a brain area that is rich in estrogen receptors and plays an important role in learning and memory (Shughrue et al., 1997; Pau et al., 1998; Gundlah et al., 2000). In addition, we set out to test the hypothesis that estrogen HRT can beneficially affect amygdala Aβ plaque density in “surgically menopausal” old females (i.e., old rhesus macaques that had undergone ovariectomy). To address these aims, we performed the following series of related studies, using archived tissues from previous unrelated rhesus macaque studies: (1) Measurement of Aβ_40_ and Aβ_42_ concentrations in the cerebral spinal fluid (CSF) to see if they reliably show age-related changes; (2) Identification of the stage of life at which Aβ plaques begin to develop within the amygdala; and (3) Examination of the effect of estradiol HRT on amygdala Aβ plaque density in old ovariectomized females, either maintained on a regular monkey chow or fed a high-fat high-sugar Western-style diet (WSD). Preliminary results were recently presented in abstract form (Urbanski et al., 2022; Appleman et al., 2023).

## Materials and methods

### Animals

Cerebrospinal fluid (CSF) and brain tissue was collected at necropsy from rhesus macaques (*Macaca mulatta*) that had been involved in various unrelated Institutional Animal Care and Use Committee approved research projects. Previously, the animals had been maintained on photoperiods comprising 12 h light and 12 h of darkness per day, and cared for in accordance with National Research Council’s *Guide for the Care and Use of Laboratory Animals*. Euthanasia was performed following an established protocol recommended by the American Veterinary Medical Association *Guidelines for the Euthanasia of Animals*, and post-mortem tissues, including CSF, were subsequently obtained through the ONPRC Tissue Distribution Program.

### Age-related changes in Aβ40 and Aβ42 in the CSF

In Experiment 1, CSF was collected at necropsy from the cisterna magna, from 44 animals (males and females), aged 8–31 years. The samples were snap frozen in liquid nitrogen, stored at −80°C, and subsequently assayed for Aβ_40_ and Aβ_42_ by Myriad RBM (Austin, TX, USA) using Luminex technology and Human CustomMAP (HMPC109). Both Aβ_40_ and Aβ_42_, concentrations were measured as single determinations. The least detectable doses (LDD) were 0.018 and 0.055 ng/ml, respectively, and the intra-assay coefficients of variation were 6.5 and 4.0%.

### Processing of brain tissue and immunohistochemistry

At necropsy, brains were flushed with 0.9% saline, and the left temporal lobes dissected and immersion fixed in 4% paraformaldehyde for 5–9 days at 4°C. Next, the tissue blocks were rinsed in 0.1 M phosphate buffer and immersed in cryoprotectant (0.1M phosphate buffer with 10% glycerol and 2% DMSO) for 1–2 days, and then immersed in cryoprotectant containing a higher of glycerol (0.1M phosphate buffer with 20% glycerol and 2% DMSO) for 3–4 days. The tissue blocks were then flash frozen in isopentane at −75°C, and stored at −20°C. Frozen coronal sections (30 μm) were subsequently cut through the amygdala and at least 4 sections (spaced 900 μm apart) from each animal were immunohistochemically stained for Aβ, to serve as biological replicates.

The immunohistochemistry was performed on free-floating amygdala sections using a standard avidin-biotin-peroxidase procedure (VECTASTAIN ABC kit; Vector Laboratories, Burlingame, CA, USA) and 3,3’-diaminobenzadine tetrahydrochloride (Sigma-Aldrich, St. Louis, MO, USA) as the chromogen. Importantly, the procedure involved incubation of sections with one of two widely used primary mouse monoclonal antibodies against Aβ, either 10D5 (Creative Biolabs, Shirley, NY, USA) or 4G8 (Biolegend, San Diego, CA, USA), at a concentration of 1:5,000 for ∼48 h at 4°C. In both cases, goat anti-mouse IgG-biotin was used as the secondary antibody (Jackson ImmunoResearch, West Grove, PA, USA).

### Age-related changes in Aβ plaque density in the amygdala

In Experiment 2, Aβ antibody 10D5 was used to immunohistochemically stain amygdala sections from 16 adult males and females, with 4 representatives from each of the following age groups: (1) < 9 years, (2) 10–19 years, (3) 20–29 years, and (4) > 30 years.

In Experiment 3, Aβ antibody 4G8 was used to immunohistochemically stain amygdala sections from 6 young (8–15 years) and 6 old (23–28 years) males that had previously served as controls in an unrelated study (Urbanski, 2017).

### Effects of diet and sex steroids on Aβ plaque density in the amygdala

In Experiment 4, Aβ antibody 4G8 was used to immunohistochemically stain amygdala sections from 12 ovariectomized females, aged 20–28 years, that had been maintained on a regular monkey chow diet. As previously described (Kohama et al., 2016), all of the animals had been ovariectomized approximately 48 months prior to necropsy and 5 of them received immediate estradiol hormone replacement therapy (HRT) in the form of subcutaneous elastomer implants; the implants were replaced every few months so as to maintain serum estradiol concentrations at levels slightly above those typically observed during the late follicular phase of the menstrual cycle (Downs and Urbanski, 2006; Sorwell and Urbanski, 2013).

In Experiment 5, Aβ antibody 4G8 was used to immunohistochemically stain amygdala sections from 14 ovariectomized females, aged 20–23 years, that had been maintained on a high-fat, high-sugar Western-style diet (WSD), starting at the time of surgery and continuing until time of necropsy approximately 30 months later (Urbanski et al., 2017; Purnell et al., 2019). Five of these animals began receiving estradiol HRT immediately in the form of subcutaneous elastomer implants that were replaced every few months, so as to maintain plasma estradiol concentrations at a mid- to late late-follicular level; in 4 other animals, the estradiol HRT was delayed for 12 months after ovariectomy as part of the original experimental design (Purnell et al., 2019).

### Image analysis

In Experiments 2 and 3, four immunohistochemically-labeled coronal amygdala sections were analyzed from each animal, while in Experiments 4 and 5, five sections were analyzed; the selected section were spaced at 900-μm intervals, centered around the central amygdala. The slide-mounted stained sections were scanned using a Leica Aperio AT2 Slide Scanner (Leica Microsystems, Buffalo Grove, IL, USA). Next, the region of interest (ROI) was selected, using QuPath 0.3.2, and exported to NIH Image J 1.53k using the Extensions feature in QuPath. In Image J, the amygdala was carefully outlined using the Polygon Tool and transformed to an 8-bit, black and white image; the threshold of this image was adjusted until the amyloid plaques became visible. Analysis of the particles was then performed using a particle size threshold set above 250 pixels; this enabled the detection of Aβ plaque density without interference of cellular membrane-bound amyloid precursor protein. The pixel area of each plaque in the amygdala region was summed to calculate the total area covered by plaques; this result was then multiplied by 100 and divided by the total area of the amygdala to obtain the percentage of the amygdala area covered by Aβ plaques. This was then averaged across the amygdala sections from each animal.

### Statistics

Results were analyzed using GB-STAT software (Dynamic Microsysyems, Silverspring, MD, USA). Spearman’s correlation was used to analyze relationships between the following: (1) Aβ_40_ and Aβ_42_ concentrations; (2) age and Aβ_40_ concentrations; (3) age and Aβ_42_ concentrations; and (4) age and Aβ_42_ to Aβ_40_ concentration ratios. Mean concentrations of Aβ_40_ and Aβ_42_ in the CSF were compared using paired Student’s *t*-test, while group means in experiments involving immunohistochemistry were compared using unpaired Student’s *t*-test.

## Results

### Aβ concentrations in the CSF

**FIGURE 1:**
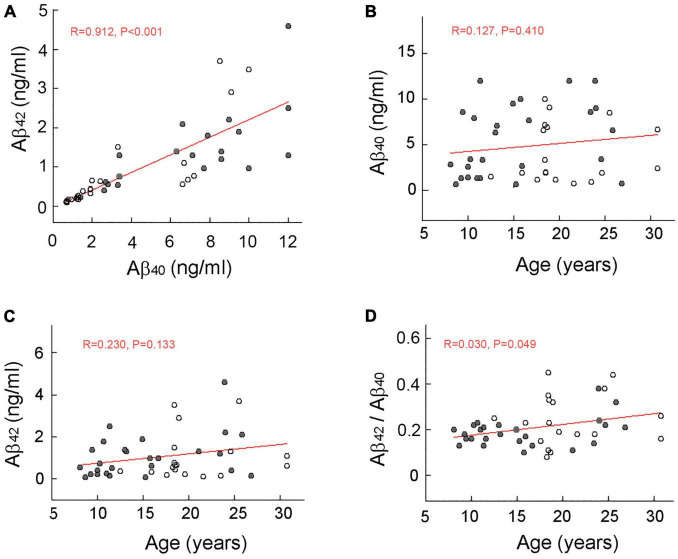
Scatterplot of amyloid beta (Aβ) concentration in the cerebral spinal fluid (CSF) of rhesus macaques. Males and females are represented by filled and open circles, respectively, and red lines depict linear regressions. A significant (*p* < 0.001) correlation between CSF concentrations of Aβ_40_ and Aβ_42_ **(A)**. No significant age-related increase in CSF concentrations of Aβ_40_ **(B)** or Aβ_42_ **(C)**. A marginally significant (*p* = 0.049) age-related increase in CSF Aβ_42_ to Aβ_40_ ratio **(D)**. The lack of a marked increase in the concentration ratio of Aβ_42_ to Aβ_40_ in the oldest animals is consistent with the observation that rhesus macaques do not develop full-blown AD during their normal lifespan.

Concentrations of Aβ_40_ and Aβ_42_ in the CSF were significantly (*p* < 0.001) correlated but showed increased divergence at higher concentrations (Figure 1A). Although no obvious age-related increase or decrease in CSF concentrations was detected in either form of Aβ (Figures 1B, C), the mean concentration of Aβ_40_ was significantly (*p* < 0.001) higher than that of Aβ_42_. The CSF concentration ratio of Aβ_42_ to Aβ_40_ showed only a marginally significant (*p* = 0.049), age-related increase (Figure 1D).

### Aβ plaque density in the amygdala across age

**FIGURE 2:**
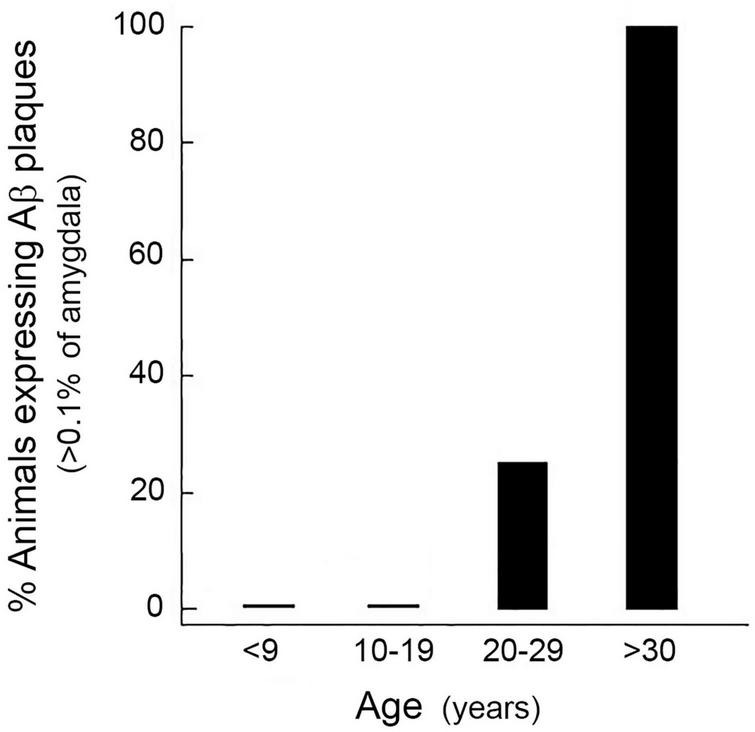
Age-related increase in the expression of amyloid beta (Aβ) plaques in the rhesus macaque amygdala. Each bar represents the mean percentage of males and females (*n* = 4, for each cohort) showing Aβ density greater than 0.1% at different decades of life. None of the animals younger than 20 years showed a significant number of Aβ plaques, whereas all of the animals older than 30 years of age did. Animals aged 20–29 years showed variable levels of Aβ expression, and so this age group served as the focus of the subsequent studies.

Aβ plaques are usually undetectable in the brains of rhesus macaques before the age of ∼20 years (Uno, 1993). In the present study, no animals younger than 20 years had Aβ plaques that exceed 0.1% of the amygdala area, whereas all of the animals older than 30 years did (Figure 2). Consequently, we considered 0.1% to represent a significant threshold in Aβ plaque development. Moreover, because animals aged 20–29 years showed variable levels of Aβ expression, this age group served as the focus of the subsequent experiments involving manipulation of diet and the sex-steroid environment.

**FIGURE 3:**
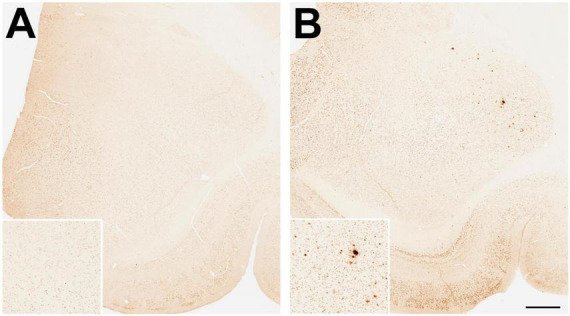
Immunohistological staining of amyloid beta (Aβ) plaques in the amygdala of young and old gonad-intact male rhesus macaques. Examples are depicted from **(A)** an 11-year-old and **(B)** a 23-year-old animal, while being maintained on a regular monkey chow. Scale bar = 100 μm for all panels; the respective insets show 2x magnified images of the central portion of the amygdala. Taken together with data shown in Figure 5A, the results demonstrate an age-related development of amyloid beta (Aβ) plaques in the amygdala.

Firstly, we wanted to corroborate the age-related increase in Aβ plaque density, and so we examined Aβ plaque density in another cohort of animals, comprising 6 young (8–15 years) and 6 old (23–28 years) males, taking advantage of available archived brain tissue from an unrelated study. The immunohistochemistry in this and all subsequent experiments utilized an alternate widely used Aβ primary antibody (4G8), instead of 10D5. As expected, none of the young males showed a significant number of Aβ plaques in the amygdala. In contrast, 50% of the older animals showed some plaques but in only one animal did the plaque density occupy > 0.1% of the amygdala area (Figures 3A, B).

### Aβ plaque density in the amygdala: effect of diet and sex steroids

**FIGURE 4:**
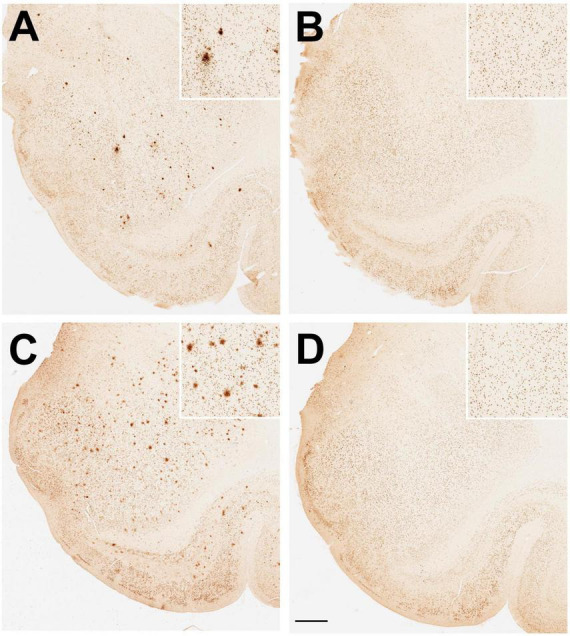
Immunohistological staining of amyloid beta (Aβ) plaques in the amygdala of aged ovariectomized female rhesus macaques. Randomized examples are depicted from **(A)** an ovariectomized (Ovx) animal and **(B)** an ovariectomized estrogen treated (Ovx + E) animal maintained on a regular monkey chow. Randomized examples are also depicted from **(C)** an ovariectomized (Ovx) animal and **(D)** an ovariectomized estrogen treated (Ovx + E) animal maintained on a high fat, high sugar Western-style diet (WSD). Scale bar = 100 μm for all panels; the respective insets show 2x magnified images of the central portion of the amygdala. Taken together with data shown in Figures 5B, C, the results demonstrate a significant effect of estradiol on amyloid beta (Aβ) plaque density in the amygdala.

**FIGURE 5:**
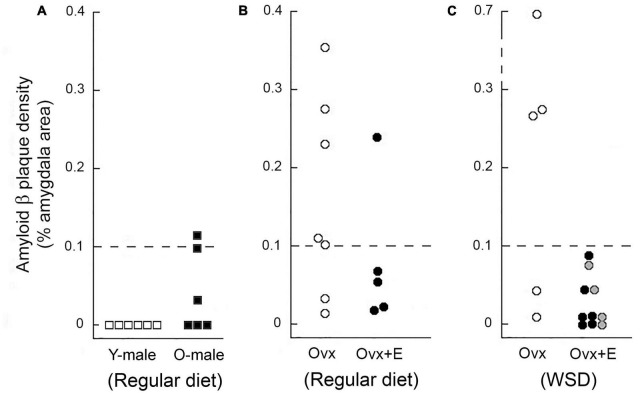
Amyloid beta (Aβ) plaque density in the amygdala of young and old male rhesus macaques maintained on a regular monkey chow **(A)**, as well as in the amygdala of aged ovariectomized females **(B)** and aged ovariectomized females that were maintained on a Western-style diet (WSD) **(C)**. Each data point represents the mean value from individuals. Data from young (Y-male) and old (O-male) males are depicted as open and black squares, respectively. Untreated ovariectomized (Ovx) animals are depicted by open circles, while data from ovariectomized animals that were subjected to estrogen HRT (Ovx + E) are depicted either by black or gray circles; in the latter case, the estrogen replacement was delayed for 12 months after ovariectomy and start of maintenance on a WSD. None of the young males showed any amygdala Aβ plaques, but 50% of the old males did; however, the density of these plaques was markedly lower than in the age-matched Ovx females. Overall, 67% of the Ovx controls showed amygdala Aβ plaque density equal or greater than 0.1%, compared to only 7% for the Ovx + E animals, thereby suggesting that loss of estrogens may play a role in promoting age-related development of Aβ plaques.

**FIGURE 6:**
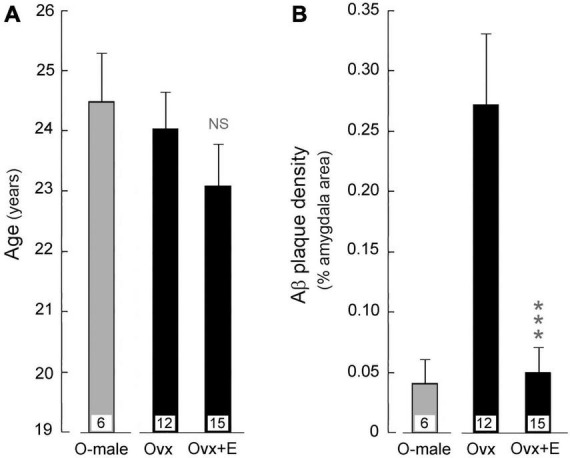
Effect of HRT on amyloid beta (Aβ) plaque density in the amygdala of aged ovariectomized female rhesus macaques; pooled data are shown from animals that were maintained on either the regular monkey chow or WSD. For reference, data from age-matched gonad-intact males (O-male) are also depicted. Mean values are represented by bars, with the number of animals per group indicated at the base of each bar; SEMs are represented by vertical lines. **(A)** The mean age of animals in the ovariectomized (Ovx) control group was not statistically (NS) different from that of the ovariectomized animals that were subjected to estrogen HRT (Ovx + E). **(B)** The mean Aβ plaque density was significantly lower in the amygdala of HRT treated ovariectomized (Ovx + E) animals compared to the untreated ovariectomized (Ovx) controls. ***p < 0.001, Student’s *t*-test. The results show that loss of estrogens likely contributes to increased Aβ plaque denisity in the amygdala, and that estradiol HRT has the potential to attenuate this increase.

Examples of positive Aβ immunohistological staining in the amygdala of aged ovariectomized female rhesus macaques are shown in Figure 4. Regardless of dietary manipulation (i.e., standard monkey chow or WSD), there was a clear difference in Aβ plaque density observed in ovariectomized (Ovx) animals compared to those receiving estradiol HRT (Ovx + E) (Figures 4A, C vs. Figures 4B, D). More than half of the Ovx animals in both dietary treatment groups showed significant amygdala expression of Aβ plaques, whereas only one Ovx + E animal did (Figures 5B, C), similar to the old males (Figure 5A). Because the Ovx animals from the two dietary studies (i.e., regular diet and WSD) had significantly different post-ovariectomy intervals (i.e., ∼48 months versus ∼30 months) it was not possible to determine with any certainty if the amygdala plaque density was significantly enhanced by a WSD. Overall, however, there appeared to be no obvious effect of diet, and so the data from the two dietary studies were pooled. Despite similarity in mean age between the Ovx and Ovx + E animals (Figure 6A), the density of Aβ plaques was significantly (*p* < 0.001) lower in the latter group and more similar to that observed in the age-matched gonad-intact males (Figure 6B).

### Discussion

Alzheimer’s disease (AD) is the most common form of dementia, affecting about 6.7 million people in the USA aged over 65 years; it also remains the fifth-leading cause of death among this age group (2023). And yet, there is still no effective cure for AD. Some pharmaceutical interventions, such as donepezil, provide temporary relief from AD symptoms, by blocking the breakdown of acetylcholine (Marucci et al., 2021; Rong et al., 2021). While others, such as memantine, regulate the activity of the excitatory neurotransmitter glutamate (McKeage, 2009; Kishi et al., 2017; Guo et al., 2020). However, none of these interventions has been shown to effectively retard the progression of the disease. More recently, aducanumab and lecanemab, two monoclonal antibody-based therapies designed to decrease the amount of Aβ plaques in the brain have become available (Sevigny et al., 2016; Shi et al., 2022; Mead and Fox, 2023; van Dyck et al., 2023). However, their effectiveness at improving cognitive function remains unclear and there is concern about potential negative side effects (Tian Hui Kwan et al., 2020; Avgerinos et al., 2021; Decourt et al., 2021).

The main goal of the present study was to investigate the therapeutic potential of endocrine-based interventions at reducing the amount of Aβ plaques in the brain, namely, estradiol-based hormone replacement therapy (HRT). Because of the conflicting evidence from clinical studies regarding the beneficial effects of estrogens at reducing the risk of AD (Tang et al., 1996; Kawas et al., 1997; Yaffe et al., 1998; Seshadri et al., 2001; Zandi et al., 2002; Shumaker et al., 2003; Yaffe, 2003; Shao et al., 2012; Pourhadi et al., 2023), we focused our studies on a pragmatic translational non-human primate model of aging.

A more proximate goal, however, was to establish if development of Aβ plaques within the brain follows a similar time course in both rhesus macaques and humans. To do this, we first examined CSF concentrations of Aβ across the first three decades of the rhesus macaque life. As expected, based on previous non-human primate studies (Gearing et al., 1996), we found the concentration of Aβ_40_ in the monkey CSF to be significantly higher than that of Aβ_42_, and agreeing with clinical studies that established Aβ_40_ to be the predominant isoforms of Aβ in humans (Gu and Guo, 2013; Dumurgier et al., 2015; Lehmann et al., 2018). Unlike AD patients (Fukumoto et al., 1996), however, the CSF concentration of Aβ_42_ or the concentration ratio of Aβ_42_ to Aβ_40_ in the oldest rhesus macaques did not show a marked increase. This finding is consistent with the observation that rhesus macaques do not develop full-blown AD during their normal lifespan, despite clearly showing an age-dependent increase in Aβ plaque density and showing mild age-related cognitive decline (without neuronal loss) (Stonebarger et al., 2020, 2021).

To test whether HRT could offer protection against development of Aβ plaques in an estrogen receptor rich part of the brain (Shughrue et al., 1997; Pau et al., 1998; Gundlah et al., 2000), we used a surgically menopausal animal model. Although female rhesus macaques clearly undergo menopause during old age (Gilardi et al., 1997; Downs and Urbanski, 2006; Sorwell and Urbanski, 2013; Luna et al., 2020), the exact age of when this occurs is quite variable between animals. In contrast, ovariectomized old (i.e., > 20 years) female rhesus macaques reliably have extremely low circulating levels of estradiol, like those observed after menopause, and thus surgically menopausal animals represent a more precise experimental model. Furthermore, we examined archived brains from two independent studies involving different diets. In one study the animals had been ovariectomized and maintained on a regular monkey chow for ∼48 months, while in the other study ovariectomized animals had been maintained for ∼30 months on a high-fat, high-sugar Western-style diet (WSD); the latter being an attempt to mimic a common diet of many post-menopausal women in the USA. In both cases, Aβ plaque density was greater in ovariectomized animals that did not receive estradiol HRT, although it should be emphasized that this Aβ plaque density was much lower than what is typically observed in the brains of AD patients (Stonebarger et al., 2020, 2021). Furthermore, the attenuated plaque density in the Ovx + E animals more closely resembled the density observed in age-matched, gonad-intact males. Because old males do not show a marked attenuation of estrogen concentrations, compared to post-menopausal females (Downs and Urbanski, 2006; Urbanski et al., 2014), this finding gives further support to the view that a lack of estrogen contributes to increased Aβ plaque density within the brain. Overall, the present results are consistent with findings from previous studies using transgenic mouse models of AD, which showed sex-differences in accumulation of Aβ plaques in the brain due to estrogen and showed that administration of estradiol was associated with lower Aβ peptide levels compared to untreated controls (Tschiffely et al., 2016; Hu et al., 2023). On the other hand, the present study was performed using a long-lived primate that more closely resembles humans in terms of its behavior, anatomy, physiology and endocrinology, and therefore represents a more translational animal model of aging, especially post-menopausal women.

It is unclear from the present experiments if the HRT prevented or delayed formation of the Aβ plaques, increased their clearance, or a combination of both. It is also unclear if a similar result would have been observed had the HRT been initiated several years after the ovariectomy. On the one hand, results from the Women’s Health Initiative study suggest that many benefits of HRT may be lost if it is delayed by several years after the onset of menopause (Lobo et al., 2014). On the other hand, it is encouraging that HRT in our WSD study showed beneficial effects even in individuals in which it had been delayed for 12 months after the ovariectomy.

Taken together, the present results demonstrate that estradiol HRT can significantly reduce Aβ plaque load in the amygdala of surgically menopausal females, even when consuming a WSD. Because of concern about adverse peripheral side effects associated with conventional HRT, it is unclear if a hormonal-based therapies would gain acceptance by post-menopausal women (German-Castelan et al., 2023). One possible way to overcome this potential problem, however, could be to rely on brain-selective prodrugs, such as 10β,17β-dihydroxyestra-1,4-dien-3-one (DHED) (Tschiffely et al., 2016), as alternatives to traditionally used estrogens. Additional research would be required to demonstrate their efficacy and safety, but they may ultimately prove to be more acceptable endocrine approaches to treating or delaying the onset of AD in humans.

## Conclusion

The results demonstrate that HRT involving estrogen can reduce Aβ plaque load in a cognitive brain region of aged non-human primates. They may therefore have clinical relevance to the treatment of AD in post-menopausal women, especially if it can be shown that HRT can also reduce Aβ plaque load in the prefrontal cortex and hippocampus. Whether used alone, or as a supplement to pharmacological and monoclonal antibody-based interventions, estrogen-based HRT could represent a more natural intervention aimed at preventing, delaying, or decreasing Aβ pathology within the brain.

## Data availability statement

The original contributions presented in this study are included in this article, further inquiries can be directed to the corresponding author.

## Ethics statement

The animal study was approved by the ONPRC Institutional Animal Use and Care Committee. The study was conducted in accordance with the local legislation and institutional requirements.

## Author contributions

HU: Conceptualization, Funding acquisition, Writing–original draft. M-LA: Methodology, Writing–review and editing. JT: Methodology, Writing–review and editing. AW: Methodology, Writing–review and editing. BN: Methodology, Writing–review and editing. RC-J: Writing –review and editing. SK: Writing–review and editing.
